# Pembrolizumab for treatment of progressive multifocal leukoencephalopathy in primary immunodeficiency and/or hematologic malignancy: a case series of five patients

**DOI:** 10.1007/s00415-021-10682-8

**Published:** 2021-07-01

**Authors:** Timo Volk, Klaus Warnatz, Reinhard Marks, Horst Urbach, Gisela Schluh, Valentina Strohmeier, Jessica Rojas-Restrepo, Bodo Grimbacher, Sebastian Rauer

**Affiliations:** 1 Department of Neurology, Medical Center – University of Freiburg, Faculty of Medicine, University of Freiburg, Breisacherstr. 64, 79106 Freiburg, Germany; 2 Department of Rheumatology and Clinical Immunology, University Medical Center Freiburg, Freiburg, Germany; 3 Center for Chronic Immunodeficiency (CCI), University Medical Center Freiburg, Freiburg, Germany; 4 Department of Hematology and Oncology, University Medical Center, Freiburg, Germany; 5 Department of Neuroradiology, University Hospital Freiburg, Freiburg, Germany; 6 Faculty of Biology, University of Freiburg, Freiburg, Germany; 7 Institute for Immunodeficiency, University Medical Center, Medical Faculty, Albert-Ludwigs-University of Freiburg, Freiburg, Germany; 8 RESIST – Cluster of Excellence 2155 to Hanover Medical School, Satellite Center, Freiburg, Germany; 9 DZIF – German Center for Infection Research, Satellite Center Freiburg, Freiburg, Germany; 10 CIBSS – Centre for Integrative Biological Signalling Studies, Albert-Ludwigs University, Freiburg, Germany

**Keywords:** PML, Pembrolizumab, PID, Hematologic malignancy, Autoimmunity

## Abstract

Progressive multifocal leukoencephalopathy is a rare opportunistic infection of the brain by John Cunningham polyomavirus in immune-compromised patients. In cases where no overt option for immune reconstitution is available [e.g., in patients with primary immunodeficiency (PID)], the disease is lethal in the majority of patients. Immune checkpoint inhibition has been applied in recent years with mixed outcomes. We present four novel patients and the follow-up of a previously published patient suffering from progressive multifocal leukoencephalopathy (PML) due to PID and/or hematologic malignancy who were treated with the immune checkpoint inhibitor pembrolizumab. In two patients with PID, symptoms improved and stabilized. One patient died because of worsening PML another of intracranial hemorrhage which was unrelated to PML or its treatment with pembrolizumab. The fifth patient suffered from PID and died of a pre-existing immune dysregulation, possibly exacerbated by pembrolizumab. The long-term follow-up of the first patient provides support for therapeutic decisions during this therapy and is the longest published clinical course of a patient with checkpoint inhibition for PML. We conclude that pembrolizumab can control PML symptoms long term in a subgroup of patients with PID, in our cases for 21 and 36 months. However, therapy must be started early because symptoms are only partially reversible. In light of severe adverse events, application of pembrolizumab is only justified if the prognosis for the individual patient is very poor.

**Supplementary Information:**

The online version contains supplementary material available at 10.1007/s00415-021-10682-8.

## Introduction

Progressive multifocal leukoencephalopathy (PML) is a rare, life threatening, opportunistic infection of the central nervous system caused by the reactivation of John Cunningham polyomavirus (JCV) almost exclusively in immunocompromised patients. Treatment focusses on restoring immunity of the affected patients since no effective antiviral therapies for JCV are available. To this end, e.g. combined antiretroviral therapy is given in HIV-infected patients or immunosuppressive drugs such as natalizumab are withdrawn in e.g., MS patients. In contrast, PML in the context of hematologic malignancy or primary immunodeficiency (PID) is lethal in the majority of cases as there are no therapeutic options for immune reconstitution [1].

The checkpoint inhibitor pembrolizumab is a monoclonal antibody disrupting interactions of programmed cell death protein 1 (PD-1) on T cells and its ligands on antigen-presenting cells. By blocking this receptor involved in negative regulation of T-cell activation, they boost immune responses. Originally, checkpoint inhibitors were developed to foster the antitumor immune response and were able to significantly improve survival in patients with metastatic melanoma [2] and others. In line with this mechanism, an association between reduced T-cell function and high PD-1 expression has also been shown in chronic viral infections such as HIV [3, 4]. Moreover, blockade of PD-1 expression in monkeys with chronic simian immunodeficiency virus infection resulted in an increase in activated virus-specific T cells, a reduced viral load, and prolonged survival of the animals [5, 6], ultimately contributing to the establishment and maintenance of HIV-1 latency [7].

Laboratory evidence of PD-1 upregulation on T cells in PML patients [8] led to the off-label use of checkpoint inhibitors in the therapy of PML patients where no other option was available to reinvigorate antiviral immunity. To our knowledge, 31 published patients with PML have so far been treated with either pembrolizumab or nivolumab. Treatment outcomes range from moderate improvement of symptoms with stabilization to death from disease progression [9–25]. To date, there are no clear prognostic factors to identify patients in whom checkpoint inhibition will be able to ameliorate PML [26].

Here, we give an update on the clinical course of one previously published patient [21] and describe four additional unpublished patients treated with pembrolizumab for PML in the context of PID and/or hematologic malignancy.

## Methods

Patients received pembrolizumab at a dose of 2 mg per kilogram body weight (unless otherwise stated) on a compassionate-use basis after informed consent was obtained. Data of patients presented in this report were retrospectively collected. For flow cytometric analyses of PD-1 expression, peripheral blood mononuclear cells were isolated from EDTA blood of patients and healthy controls by Ficoll density gradient centrifugation following Standard protocols. Staining for PD-1 was performed on freshly isolated PBMCs or whole blood. Staining was performed for 15 min at 4°. In case of whole blood staining, red blood cell lysis was performed, following staining, using OptiLyse B (Beckman Coulter). Data were acquired using an LSR Fortessa (BD Biosciences) or FACS CANTO II (BD Biosciences). Data were analyzed using FlowJo Software (Treestar). Cells were stained with antibodies against CD45RA (PE-Cy7, HI100), CD3 (APC-H7, SK7), CD 4 (FITC, RPA-T4, all above from BD Pharmingen, Heidelberg, Germany), CD8 (PerCP-Cy5.5, RPA-T8, Invitrogen, Carlsbad, USA), CD3 (PerCp-Cy5.5, SK7), CD4 (PE-Cy7, RPA-T4), CD8 (PE, SK1), CD45 (BV421, HI30), CD45RA (APC-Cy7, HI100) and PD-1 (APC, EH12.2H7, all above from Biolegend, San Diego, USA). Additional laboratory analysis and magnetic resonance imaging (MRI) were performed as part of standard clinical care.

## Results

**Fig. 1:**
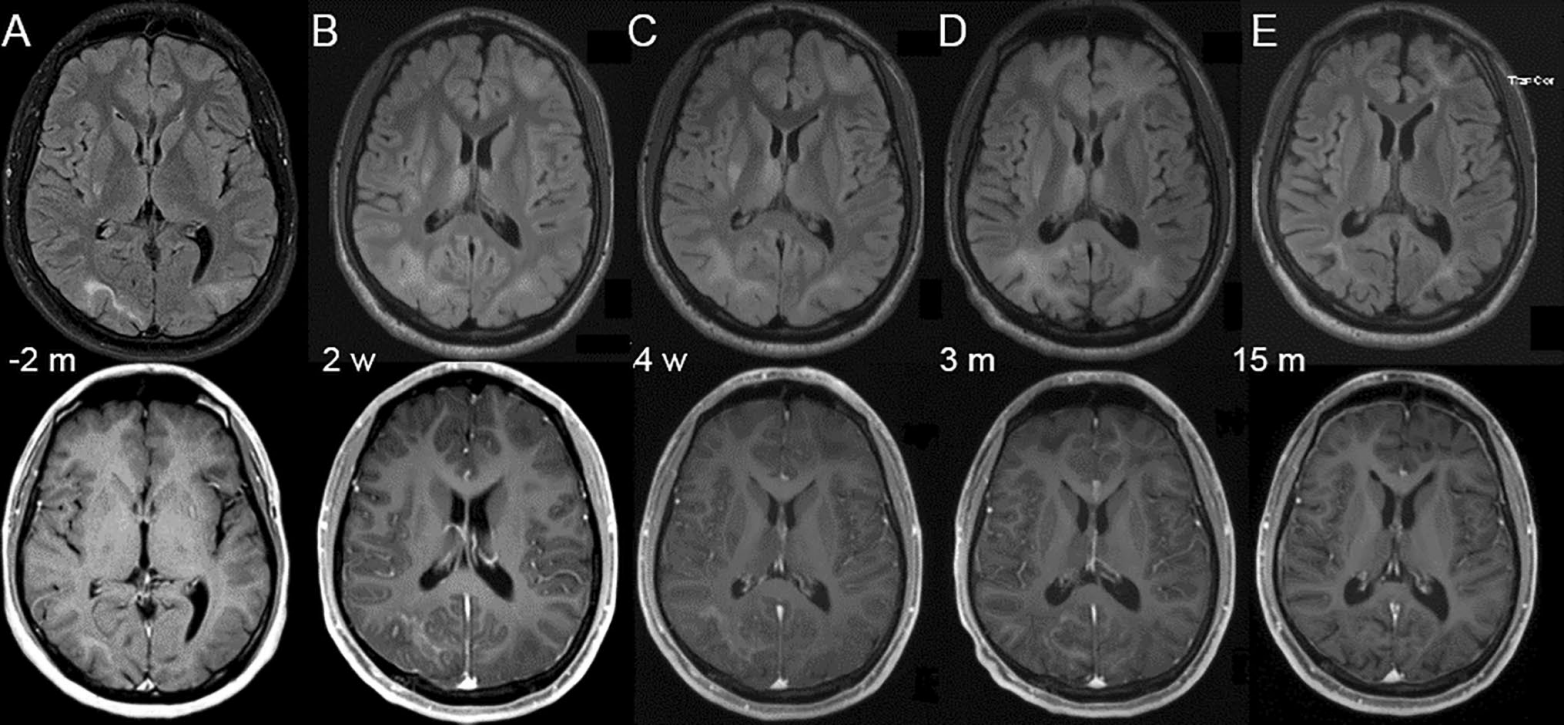
Transiently contrast-enhancing lesion in Patient 1. **A** Two months before treatment, bi-occipital lesions with minimal contrast enhancemend on the right as well as a lesion in the globus pallidus are visible. **B** Lesions progress after treatment, a new lesion in the left frontal lobe, as well as bi-thalamic lesions are visible. Contrast enhancement is increased in the right occipital lesion. **C** Lesion in the thalamus is less intense, otherwise stable MRI. **D** + **E** Declining intensities of lesions, no contrast enhancement is detectable 15 months after initial treatment. Top row: *FLAIR* (Fluid-attenuated inversion recovery) MRI sequences, bottom row: *MP RAGE* (magnetization-prepared 180 degrees radio-frequency pulses and rapid gradient-echo sequences) MRI sequences post gadolinium application. *m* month,* w* week

Patient 1: a 21-year-old male patient with the diagnosis of CD40-ligand deficiency (MIM #308,230) was referred to us with a diagnosis of PML after progressive visual impairment over the past two months. MRI showed bi-occipital lesions on FLAIR (fluid-attenuated inversion recovery) sequences and a lesion in the globus pallidus on the right. JCV-PCR from cerebrospinal fluid (CSF) was positive with 471 copies per milliliter. One week after the first dose of pembrolizumab, he developed a maculopapular rash, which dissolved without further specific treatment and was considered an immune‐related adverse event. After another week, the patient was in a stable clinical condition. MRI showed mild contrast enhancement (Fig. 1) as sign of possible immune reconstitution inflammatory syndrome (IRIS), the JCV-PCR was still positive (but < 500 copies/ml). Another 2 weeks later the patient developed short term memory impairment and mild disorientation. Contrast enhancement in MRI remained stable while FLAIR lesions showed progression. The patient received a second dose of pembrolizumab 5 weeks after the first one. Subsequently the disorientation and memory impairment abated, JCV-PCR was negative and MRI showed stable lesions with persistent contrast enhancement. A third dose of pembrolizumab was administered 8 weeks after the first infusion. In follow-up visits the patient reported minimal improvement of visual impairment but showed no signs of cognitive impairment. He was able to resume his studies. MRI showed constant FLAIR lesions without contrast enhancement and JCV-PCR remained negative at follow-up 15 months after the first dose of pembrolizumab. A graphical overview of the clinical course is given in Figure S1 in this article’s Online Repository.

**Fig. 2:**
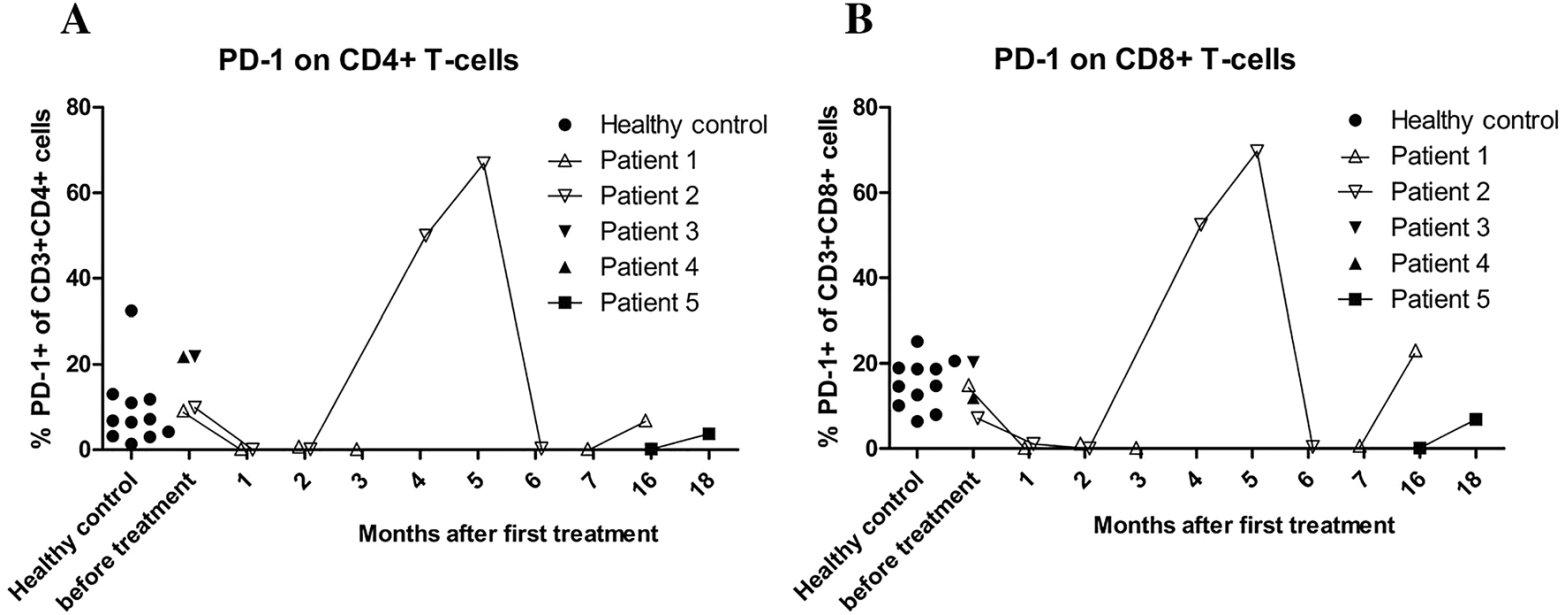
Patients show reduced expression of PD-1 on T cells after Pembrolizumab treatment. PD-1 expression is within normal range on CD4 + (A) and CD8 + (B) T cells in Patient 1 and 2 before treatment. Patient 3 and 4 show normal expression on CD8 + (B) and high normal expression on CD4 + T cells before treatment. All patients show a reduction of PD-1 expression after treatment. An increase is seen on T cells from Patient 2 four and five months after the first infusion during severe autoimmune adverse reactions. Cells from Patient 5 were analyzed only 16 and 18 months after commencement of therapy and show low to low normal expression of PD-1

Patient 2: A 45-year-old woman presented with fluctuating hypesthesia of the left leg for a few weeks. The patient was on immunoglobulin replacement therapy for common variable immunodeficiency (CVID) with immune dysregulation including enteropathy, autoimmune cytopenia and interstitial lung disease. For enteropathy, she received budesonide. Rituximab had been administered 15 years before presentation for immune thrombocytopenia and hemolytic anemia. MRI showed a parietooccipital FLAIR lesion on the right hemisphere without contrast enhancement. A stereotactic biopsy was performed after extensive testing of CSF (including negative JCV-PCR). PML was diagnosed based on a strongly positive JCV-PCR from the biopsy and pembrolizumab was administered. Within days, symptoms progressed with mild hemiparesis on the right. MRI showed mild contrast enhancement as sign of IRIS. After a complete stabilization with only mild persistent hypesthesia and no signs of worsening enteropathy, the patient received a second dose of pembrolizumab after 3 weeks. Further treatment was withheld because of stabilization after the second dose and the potential reactivation of the pre-existing autoimmune condition. After intermittent diarrhea and leukopenia, a third dose was given 4 months after the first dose because of mild progression of FLAIR lesions on MRI without contrast enhancement concomitant with increase in PD-1 expression on T cells after an initial decrease (Fig. 2). Subsequently, the patient developed severe leukopenia requiring corticoid therapy and administration of granulocyte colony-stimulating factor and the diarrhea worsened. Additionally, there was laboratory evidence of autoimmune hepatitis. Because of newly developing cognitive deficits with neglect and progression of the parietooccipital FLAIR lesion and after discussion with the patient and relatives a fourth dose of pembrolizumab was administered with a reduced dose of 0.5 mg per kilogram body weight after leukopenia, diarrhea and hepatitis had abated over a period for approximately 1 week. PD-1 expression on T cells decreased again after the fourth dose but leukopenia reoccurred and she developed a pneumonia. In light of the clinical deterioration and the desperate situation the patient opted for palliative care and subsequently died from bleeding due to severe thrombocytopenia 6 months after the beginning of pembrolizumab treatment. A graphical overview of the clinical course is given in Figure S2 in this article’s Online Repository.

Patient 3: A 78-year-old man presented with slowly progressing hemianopia over a few weeks. He had received chemotherapy including rituximab for the treatment of a diffuse large B-cell lymphoma (DLBCL) 1 month prior to development of the symptom. MRI showed a large right occipital FLAIR lesion without contrast enhancement. JCV-PCR of CSF was positive (< 500 copies/ml). Pembrolizumab was administered. Three weeks later, the patient reported worsening vision of the left eye and a tumor of the iris was identified. Additionally, he showed several tumors of the arm and leg suspicious of enlarged lymph nodes. A biopsy of one lymph node showed a relapse of the lymphoma. The occipital FLAIR lesion had progressed without contrast enhancement. However, a left sided contrast-enhancing lesion had developed, suspicious of lymphoma. While being evaluated for further therapeutic options regarding the lymphoma, he developed a coma due to an extensive intracranial hemorrhage on the left hemisphere under full anticoagulation therapy for atrial fibrillation. He died a few days later under palliative care. A graphical overview of the clinical course is given in Figure S3 in this article’s Online Repository.

Patient 4: A 45-year-old man presented with progressing dysarthria, impaired vision and attention deficit for 2 weeks. He suffered from a combined immunodeficiency due to a deleterious compound heterozygous mutation in *DOCK8* (dedicator of cytokinesis 8, MIM # 243,700), symptomatic with recurrent tumors of the skin, two episodes of meningitis in childhood, and mild lymphopenia (including at presentation to our clinic). An MRI showed bi-occipital FLAIR lesions without contrast enhancement and after positive JCV-PCR in the CSF, the diagnosis of PML was made. Pembrolizumab was administered for the first time. Relatives described decreasing dysarthria after the first dose. After another 2 weeks the patient developed non-fluent aphasia, attention deficit, further deterioration of his vision and right-sided hemiparesis. JCV-PCR from the CSF rose to 10.000 copies/ml and FLAIR lesions progressed with contrast enhancement at the borders. A second dose of pembrolizumab was administered. After being discharged to a rehabilitation clinic with stable deficits, the patient developed abdominal pain without diarrhea. No clear etiology for this pain (including autoimmune enteropathy, pancreatitis or hepatitis) could be established, and the pain subsided after antibiotic treatment. Symptoms of PML had progressed to severe spastic hemiparesis, JCV-PCR from the CSF was again positive with 68.500 copies/ml and the MRI showed a progression of lesions to the thalamus. Since PML lesions progressed with only mild maculopapular rash as sign of an autoimmune adverse reaction a third dose of pembrolizumab was administered. One week later the patient had two generalized epileptic seizures and fell into a persistent coma. Palliative care was initiated and the patient died a few days later. A graphical overview of the clinical course is given in Figure S4 in this article’s Online Repository.

**Fig. 3:**
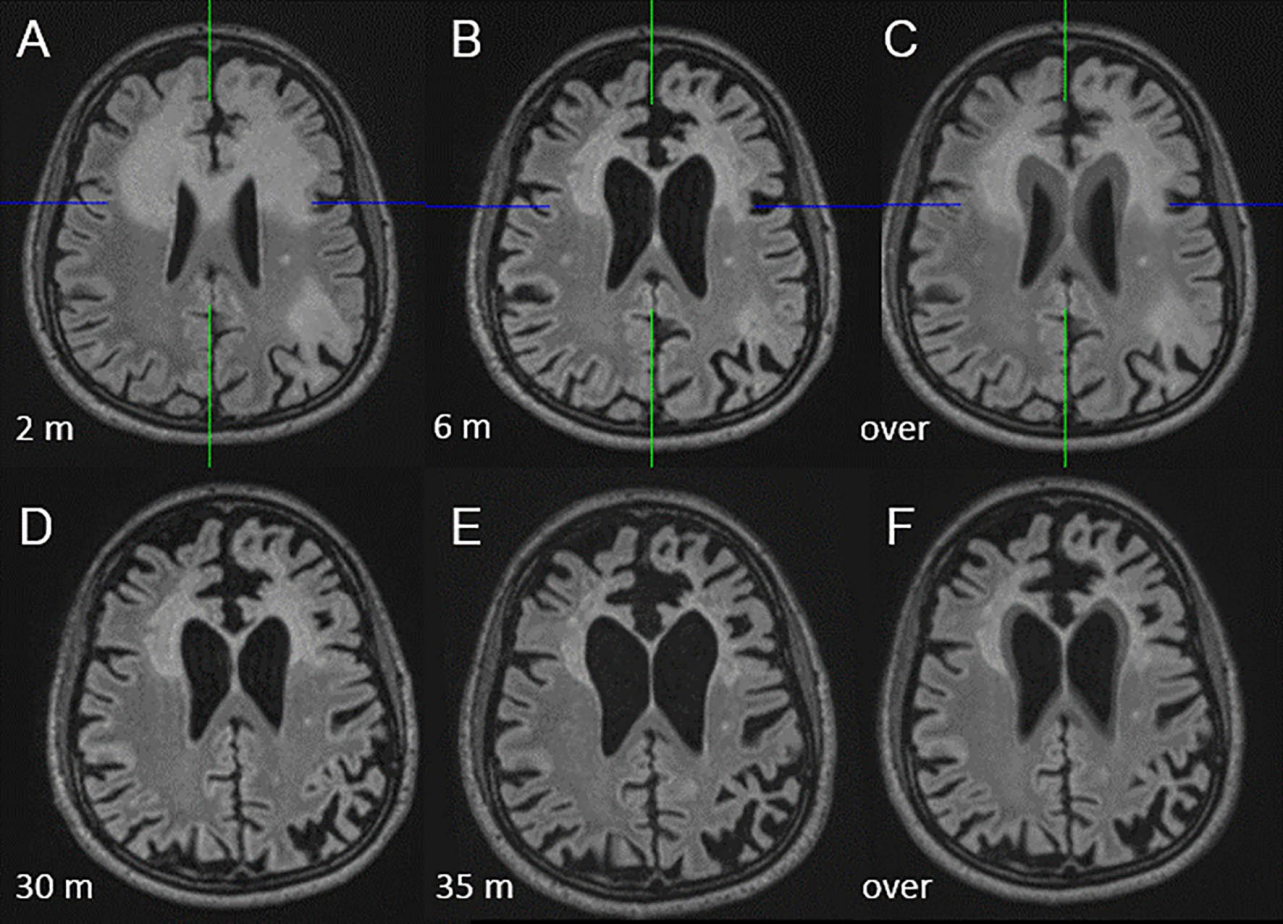
Shrinking PML lesion during therapy and progressive atrophy in Patient 5. FLAIR images showing the largest lesion extension (**A**) after two months. **B** Shrinkage of the lesion four months later (B), previously published in [21]. (D) and (E) showing progressive tissue atrophy between 30 and 35 months after commencement of pembrolizumab. **C** is an overlay of A and B, (F) is an overlay of (D) and (E) with 50% transparency each. *m* indicating months after the first administration of pembrolizumab, *over* overlap

Patient 5: A 49-year-old patient presented to us with aphasia, ataxia, apraxia and difficulties in memory. This patient’s initial clinical course has already been published [21]. In short, he was diagnosed with PML by positive JCV-PCR (1,150 copies/ml) in CSF and left-hemispheric FLAIR lesions on MRI. He had previously been diagnosed with CVID and had received Rituximab for DLBCL. Because of worsening of symptoms (mutism) and progressing lesions on MRI pembrolizumab was administered. The patient was subsequently able to speak again and JCV-PCR was negative except for 1 analysis when intervals of dosing were extended from 2 to 4 weeks. Contrast enhancement was detected on MRI after the 6th infusion compatible with IRIS, but the patient showed no corresponding symptoms. After the 8th infusion the patient had self-limiting diarrhea for a few days. Because of stable symptoms and MRI lesions dosing intervals were extended to monthly intervals after the 7th infusion. Before the 10th infusion, JCV-PCR was positive, therefore pembrolizumab was then given every 2 weeks and after negative PCR every 3 weeks. After the 24th infusion Transaminases were elevated and therefore pembrolizumab was halted. JCV-PCR was positive for 4 months (2 months after pembrolizumab was halted), but was negative again without further administration of pembrolizumab. Lymphopenia had abated without specific treatment. Thirty-nine months after onset of symptoms and 36 months after the first infusion, the patient is stable with severe aphasia, cognitive impairment and is dependent on care from his wife. However, during treatment he showed incremental improvement of aphasia and interaction (ability to utter needs with incomplete sentences and comprehension and execution of simple commands) and ataxia (ability to walk on his own and recently successful attempts to ride a bike with support). MRI shows progressive tissue atrophy without signs of active PML (Fig. 3). JCV-PCR remained negative. A graphical overview of the clinical course is given in Figure S5 in this article’s Online Repository.

## Discussion

Without efficient antiviral therapy, the mainstay of PML therapy is immune reconstitution. In conditions where reversal of immune suppression is not achieved mortality is high. This is demonstrated by a median survival of 2 months in a case series of patients with in hematologic malignancies and stem-cell transplantation [27]. Checkpoint inhibition has been applied so far in 11 cases with nivolumab therapy and 20 cases with pembrolizumab therapy (including our previous report) with varying results [9–25]. Here were report on a series of 4 novel patients with PML in the context of PID or hematologic malignancy treated with pembrolizumab and follow-up data on one previously reported patient [21].

Two patients in this cohort showed long-term stabilization judged by regression of MRI lesions and ultimately negative JCV-PCR from CSF. Symptoms improved moderately but patients still suffer from significant sequelae from PML. This is representative of other published patients with good clinical outcomes, in whom deficits were stable or showed moderate improvement [9, 10, 15, 23, 25]. Only one patient with frontal lobe syndrome due to PML showed complete independence in daily life after treatment with IL-2 and pembrolizumab [17]. The death of Patient 3 due to intracranial hemorrhage—most likely in a lesion of his recurrent lymphoma under anticoagulation therapy—rather demonstrates the aggressive disease causing the underlying immunodeficiency than failure of pembrolizumab. Death from other causes (mainly progressive cancer) is also reported in other PML patients treated with nivolumab or pembrolizumab [13, 17].

In Patient 4 disease progression could not be stopped, demonstrated by rising copy numbers of JCV-DNA in CSF and increasing lesions on MRI. Similar catastrophic clinical courses have been published previously, including a young patient who was diagnosed early after mild symptoms developed and had low copy numbers of JCV-DNA in CSF at diagnosis [10, 12, 18, 20, 22]. This challenges the concept that the disease can be controlled if treatment was commenced early and raises the question, if the underlying immunodeficiency itself is a prognostic factor. Medrano et al*.* hypothesize that the fatal outcome in all of their three patients was due to the cyclosporine given to prevent kidney transplant rejection [18]. However, a patient who responded to nivolumab also received cyclosporine one month prior to developing PML [23]. In our case the patient suffered from a combined immunodeficiency due to mutations in *DOCK8* known for difficulties in the control of certain viral infections, so that even the blocking of PD1-mediated inhibition may have not been sufficient to overcome the T-cell deficiency which are also known to have a migratory defect in *DOCK8*-deficient patients, uncurable by checkpoint blockade.

Therefore, a careful evaluation of the cellular immune status may be informative of the potential outcome. Severe and especially inborn lymphopenia or functional T-cell deficiency might dampen the therapeutic success of checkpoint inhibition. Along this line, two groups applied IL-2 before administration of pembrolizumab in three patients. One patient had a good outcome, the other two died of other diseases (lymphoma and pneumonia) [11, 17]. Patients with a desirable outcome in our report did not show lymphopenia before therapy (Patient 1) or showed rising lymphocyte counts during therapy (Patient 5). Regarding lymphocyte subsets, B-cell lymphopenia was most prominent in our cohort (Table 1) and in a recently published series of six cases [25]. However, B-cell counts did not serve as a prognostic marker exemplified by Patient 4 having normal B-cell counts and no response to therapy. T-cell subset analyses showed an expansion of activated memory T cells while naïve populations were reduced – often seen in patients with PID—and did not differ between patients with regard to their outcome (Table S1). Pawlitzki et al*.* propose analyzing lymphocyte subsets since they found higher frequencies of progenitor-exhausted T cells in a patient with good clinical response to pembrolizumab and higher frequencies of terminally exhausted T cells in a patient without response to pembrolizumab [20]. Progenitor and terminally exhausted T cells have not been analyzed in out cohort.

**Table 1:** Patient characteristics, laboratory data and clinical course

Patient Sex and age at presentation underlying condition	Cell counts before first dose [/µl]		T-cell subsets before first dose^†^	JCV PCR in CSF at diagnosis [copies/ml]	JCV PCR in CSF during treatment	Start of pembrolizumab after onset of PML symptoms	IRIS	Possible autoimmune adverse reactions	Outcome
Patient 1 Male, 21 years CD40-ligand deficiency	Leuko Lympho CD3 CD19 CD4 CD8	6810 **3070 ↑** **2442 ↑** 479 457 **1577↑**	Expansion of activated CD8 + T cells and early and late CD8 + T cells	471	**Negative** after 2 infusions 8 weeks after first infusion	10 weeks	Possibly, CE after first infusion, confusion 4 weeks later	Maculopapular rash	Stable, significant sequelae (cortical blindness)
Patient 2 Female, 45 years CVID	Leuko Lympho CD3 CD19 CD4 CD8	4150 **200 ↓** **183 ↓** **0 ↓** **142 ↓** **37 ↓**	Reduction of naive CD4 + T cells Expansion of memory CD8 + T cells	Negative in CSF, PCR from brain biopsy positive	Not applicable (negative in CSF at diagnosis)	Approx. 3 months	Likely, CE 4 days after first infusion, concomitant worsening of symptoms	Recurrent diarrhea, severe pancytopenia	Death, possibly due to autoimmune complications (bleeding, severe thrombocytopenia), initially improved regarding PML symptoms
Patient 3 Male, 78 years DLBCL	Leuko Lympho CD3 CD19 CD4 CD8	7610 **864 ↓** 778 **0 ↓** 500 265	Not done	Positive (< 500)	**Negative** after 1 infusion 4 weeks after first infusion	< 4 weeks (precise onset unknown)	No	None	Death, due to unrelated disease, stable regarding PML symptoms
Patient 4 Male, 45 years CID due to DOCK8 deficiency	Leuko Lympho CD3 CD19 CD4 CD8	7580 **861 ↓** **555 ↓** 195 **261 ↓** 282	Reduction of naive CD4 + T cells and naive CD8 + T cells	500	**Rising** to 68.500 after 2 infusions, 6 weeks after first infusion	2 weeks	No, CE at borders of PML lesion concomitant with rising JCV in CSF suggestive of advancing PML	maculopapular rash	Death due to rapidly worsening PML
Patient 5 Male, 49 years CVID, DLBCL	Leuko Lympho CD3 CD19 CD4 CD8	5000 **234 ↓** **111 ↓** **0 ↓** **40 ↓** **62 ↓**	Reduction of naive CD4 + and CD8 + T cells Expansion of activated T cells, antigen experienced CD4 + T cells and early CD8 + effector cells	1150	**Rising** to 252.500 after first infusion **Negative** after 5 infusions 9 weeks after first infusion Subsequently **Transiently positive** with low copy numbers§	4 months	Unlikely, CE 9 weeks after first infusion without corresponding symptoms	Transient mild diarrhea, Transaminitis	Stable, initial improvement of attention and speech, severe sequelae

*JCV* John Cunningham polyomavirus, *CSF* Cerebrospinal fluid, *IRIS* immune reconstitution inflammatory syndrome, *CE* contrast enhancement on MRI, *CVID* common variable immunodeficiency, *Leuko* Leukocytes, *Lympho* Lymphocytes, *DLBCL* diffuse large B-cell lymphoma, *CID* combined immunodeficiency

Bold numbers indicate values above (↑) or below (↓) reference values [30, 31]

^†^Table depicting values of subsets and subset defining markers in Table S1 in this article’s Online Repository

PD-1 expression on T cells from peripheral blood from our patients was within the range of healthy controls. Pembrolizumab administration abrogated PD-1 expression successfully on circulating T cells in all patients and therefore was not predictive for outcome or negativity of JCV-PCR (Fig. 2). Patient 1 showed an increase in PD-1 expression 14 months after the last infusion. JCV-PCR was negative 13 months after the last infusion, indicating that viral control is possible even after the effect of pembrolizumab wears off. In Patient 5 PD-1 expression was still decreased 4 months after the last infusion. JCV-PCR was transiently positive at that point. Patient 2 showed clinical and MRI progression of the disease after an initial response. While PML was progressing, PD-1 expression first increased and then decreased again. This indicates that a reduction in PD-1 expression might be prerequisite for viral control at beginning of therapy but does not strictly correlate with viral control or symptoms of PML.

In line with these findings, a clinical response to pembrolizumab was not correlated to PD-1 suppression in a cohort of eight patients published by Cortese et al*.* [10]. However, in vitro T-cell reactivity to JCV peptides was only detected in patients who also responded to therapy. This indicates that pembrolizumab might only be able to support a pre-existing immune response to JCV.

Although pre-existing autoimmune diseases not necessarily exclude treatment with checkpoint inhibitors in patients with cancer, clinical experience regarding exacerbation of already established autoimmune conditions after application of checkpoint inhibitors is rare. Nevertheless, autoimmune phenomena (e.g., diarrhea, pneumonitis) as a result of checkpoint inhibition is frequently being observed in cancer patients. Autoimmune adverse reactions, such as myositis, rashes and worsening of a pre-existing digestive tract granulomatosis, have been reported in PML patients [14, 23, 25]. None of the previously published PML patients experienced autoimmune cytopenia after treatment with pembrolizumab or nivolumab, despite one of the patients suffered from autoimmune thrombocytopenia before development of PML [10, 16, 20, 24]. In contrast, Patient 2 showed marked exacerbation of pre-existing cytopenia with only transient response to granulocyte colony-stimulating factor and corticosteroid therapy and enteropathy, which was not prevented by increased budesonide prophylaxis. She developed a pneumonia during leukopenia and ultimately died from bleeding due to thrombocytopenia likely triggered by pembrolizumab after wishing for no further treatment. Given her significant initial improvement of symptoms caused by PML, our prophylactic options for secondary immune exacerbation must be improved and in the absence of suitable prophylaxis benefits of checkpoint inhibition must be strictly weighed against possible risks in patients with pre-existing autoimmune conditions and patients need to be plainly informed about the potential risk.

IRIS after checkpoint inhibition has rarely been reported [13, 21, 22, 24]. Four of our five patients showed contrast enhancement on MRI after initiation of pembrolizumab, but only Patient 2 had concomitant worsening of symptoms. Paralleled to his clinical deterioration patient 4 showed marked contrast enhancement on the borders of the lesions on MRI also in line with progressing PML. In HIV-infected patients signs of IRIS have been proposed to indicate better prognosis [28, 29], results from our patients and previously published patients are ambiguous.

Given the short history of pembrolizumab therapy in PML, many therapeutic decisions have to be made without precedence. Observations from patients in this report including a unique 38-month follow-up add substantially to previously published data: (i) Patients with certain pre-existing T-cell deficiencies may not respond to pembrolizumab therapy for PML. (ii) The number of infusions required for disease control may vary between patients and (iii) patients with poorly controlled autoimmune disorders may not be able to receive the minimal effective dose of pembrolizumab due to severe life-threatening exacerbation of autoimmune adverse reactions. (iv) Fluctuating results from JCV-PCR may not necessitate reinstallation of pembrolizumab.

## Conclusion

For a subset of patients with immunodeficiency due to PID and/or hematologic malignancy pembrolizumab treatment can stop disease progression while symptoms may improve only moderately. Further studies must carefully define subset of patients, in whom checkpoint inhibition is a valuable treatment option. Laboratory analyses of in vitro T-cell reactivity to JCV [10] or detection of progenitor-exhausted T cells are candidate surrogates for an immune state which can be enhanced by checkpoint inhibition. Pre-existing autoimmunity is a severe contraindication which requires a careful and consented decision if therapy is justified on an individual basis. Clinical courses of treated patients must be closely monitored and analyzed to reach optimal outcome.

## Supplementary Information

Below is the link to the electronic supplementary material.

Supplementary file1 (PDF 930 KB)

## Data Availability

Not applicable.
