# Supplementary material for: Pembrolizumab for treatment of progressive multifocal leukoencephalopathy in primary immunodeficiency and/or hematologic malignancy: a case series of five patients
Source: J Neurol. 2021 Jul 1;269(2):973–81. doi: 10.1007/s00415-021-10682-8 (PMC8782776; doi:10.1007/s00415-021-10682-8)
Supplement: Supplementary file 1 — Supplementary file1 (PDF 930 KB) [file 415_2021_10682_MOESM1_ESM.pdf]

Supplementary appendix

This appendix is provided by the authors give additional information to the main article entitled *Pembrolizumab for treatment of progressive multifocal leukoencephalopathy in primary immunodeficiency and/or hematologic malignancy: a case series of 5 patients*.

**Table S1. T cell subsets before therapy**					
T cell subset definition	Reference range [%] ‡	Patient 1	Patient 2	Patient 4	Patient 5
Activated T cells [% of DR+ in CD3+]	4 – 18	**28,6 ↑**	10,4	10,9	**37,7 ↑**
Activated CD4 T cells [% of DR+ in CD3+CD4+]	3 – 12	6,9	9,2	11,3	**21,8 ↑**
Naive CD4 T cells [% of CD45RA+ in CD3+CD4+]	21 – 58	52,6	**9,1 ↓**	**2,8 ↓**	**10,4 ↓**
Antigen experienced CD4 T cells [% of CD45RO+ in CD3+CD4+]	35 – 73	43,6	**86,2 ↑**	**96,5 ↑**	**87,4 ↑**
Activated CD8 T cells [% of DR+ in CD3+CD8+]	4 – 32	**38 ↑**	14,7	7,2	**45,6 ↑**
Naive CD8 T cells [% of CD28+CD27+CD45RA+ in CD3+CD8+]	23 – 73	**18,24 ↓**	23,88	**1,54 ↓**	**15,56 ↓**
Memory CD8 T cells [% of CD28+CD27+CD45RA- in CD3+CD8+]	13 – 43	20,62	**58,94 ↑**	56,67	31,21
Early effector CD8 T cells [% of CD28-CD27+ in CD3+CD8+]	3,4 – 17	**19,4 ↑**	7,4	6,3	**21,4 ↑**
Late effector CD8 T cells [% of CD28-CD27- in CD3+CD8+]	1,6 – 36	32,8	4,8	16	20,8

‡ Reference values from [1, 2]

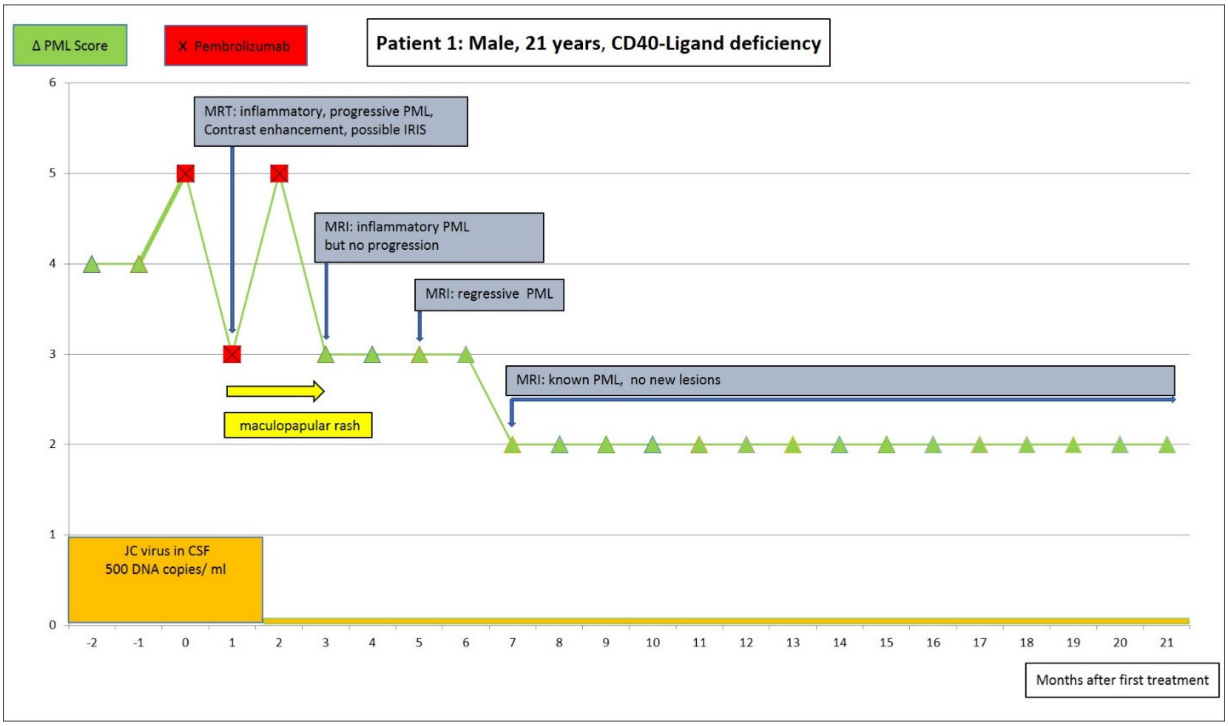

Figure S1: Clinical course of Patient 1
The PML Score is a clinical score comprised of the NIH stroke scale in addition to assessing vestibular function, dysphagia, executive function and memory previously proposed by Gasnault *et al.* [3] ranges are from 0 (no symptoms) to 60 (severe deficit)

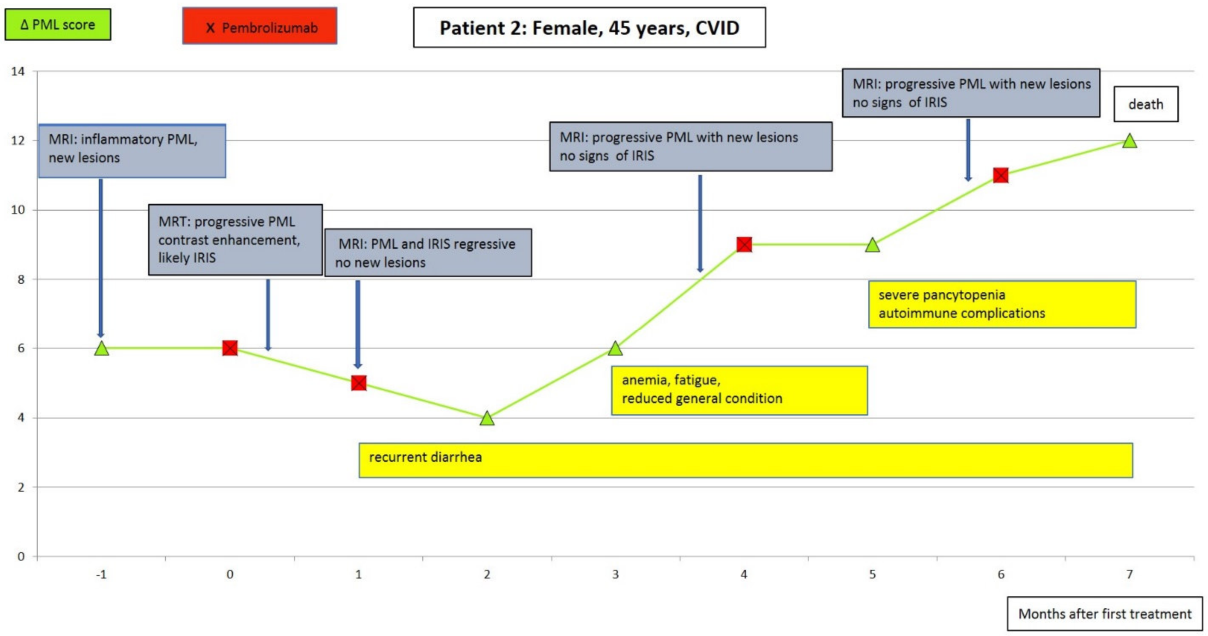

Figure S2: Clinical course of Patient 2

The PML Score is a clinical score comprised of the NIH stroke scale in addition to assessing vestibular function, dysphagia, executive function and memory previously proposed by Gasnault *et al.* [3] ranges are from 0 (no symptoms) to 60 (severe deficit)

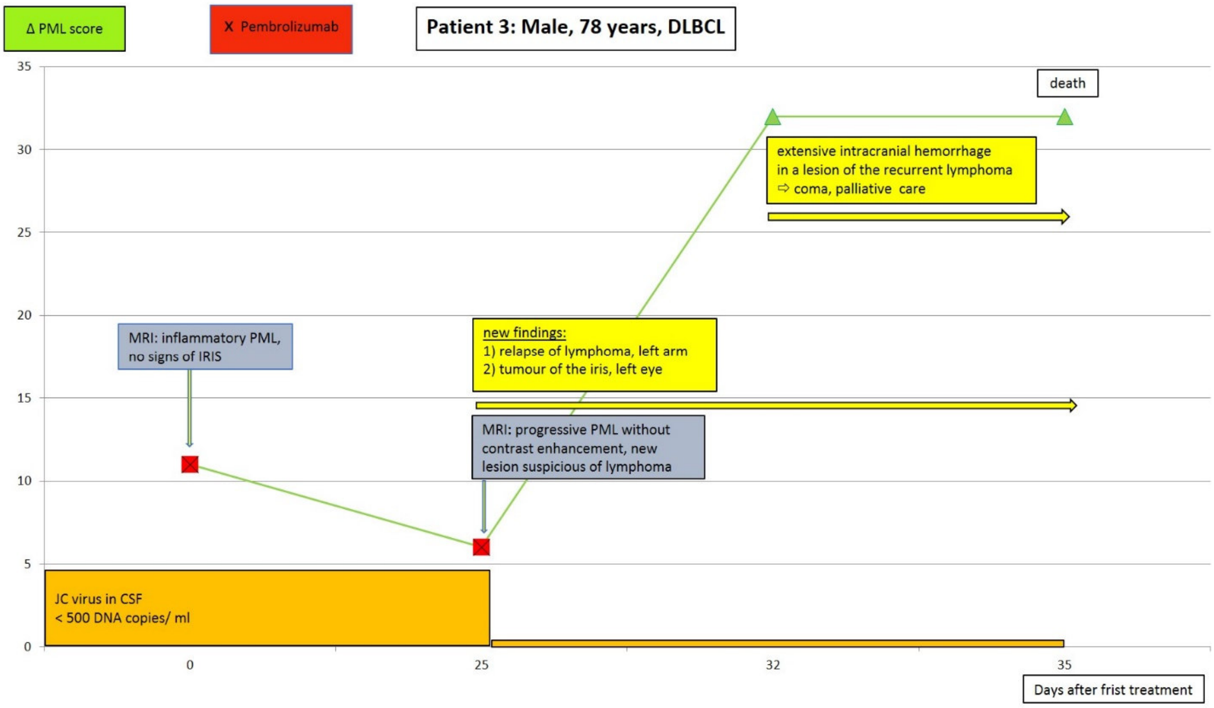

Figure S3: Clinical course of Patient 3
The PML Score is a clinical score comprised of the NIH stroke scale in addition to assessing vestibular function, dysphagia, executive function and memory previously proposed by Gasnault *et al.* [3] ranges are from 0 (no symptoms) to 60 (severe deficit)

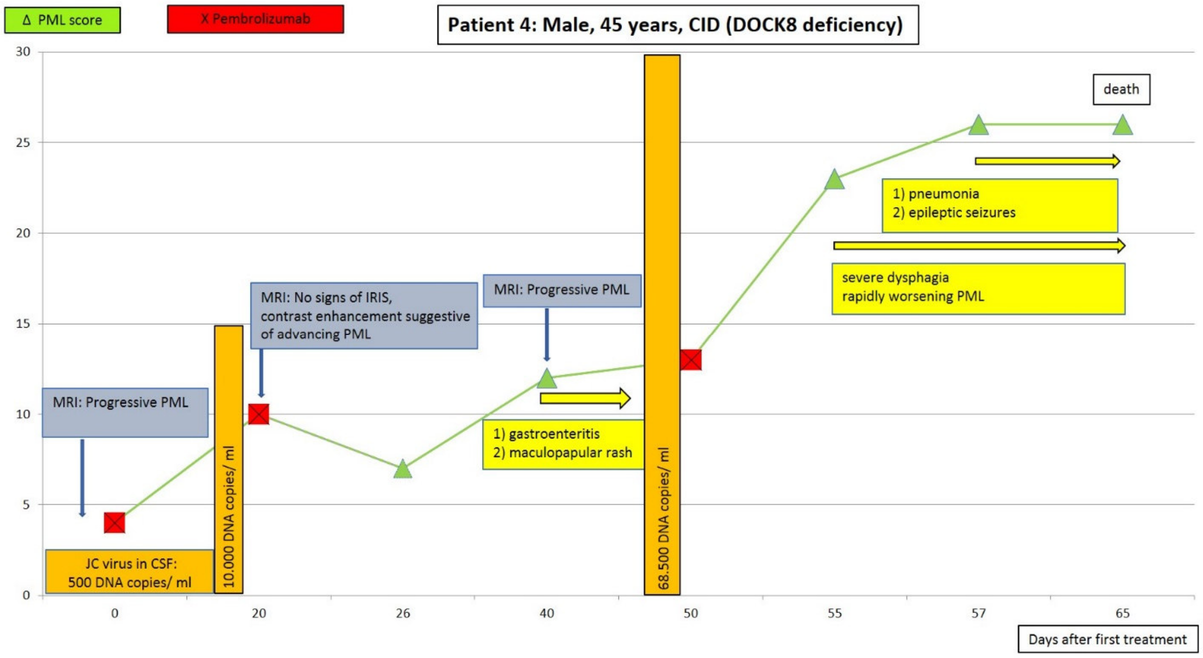

Figure S4: Clinical course of Patient 4
The PML Score is a clinical score comprised of the NIH stroke scale in addition to assessing vestibular function, dysphagia, executive function and memory previously proposed by Gasnault *et al.* [3] ranges are from 0 (no symptoms) to 60 (severe deficit)

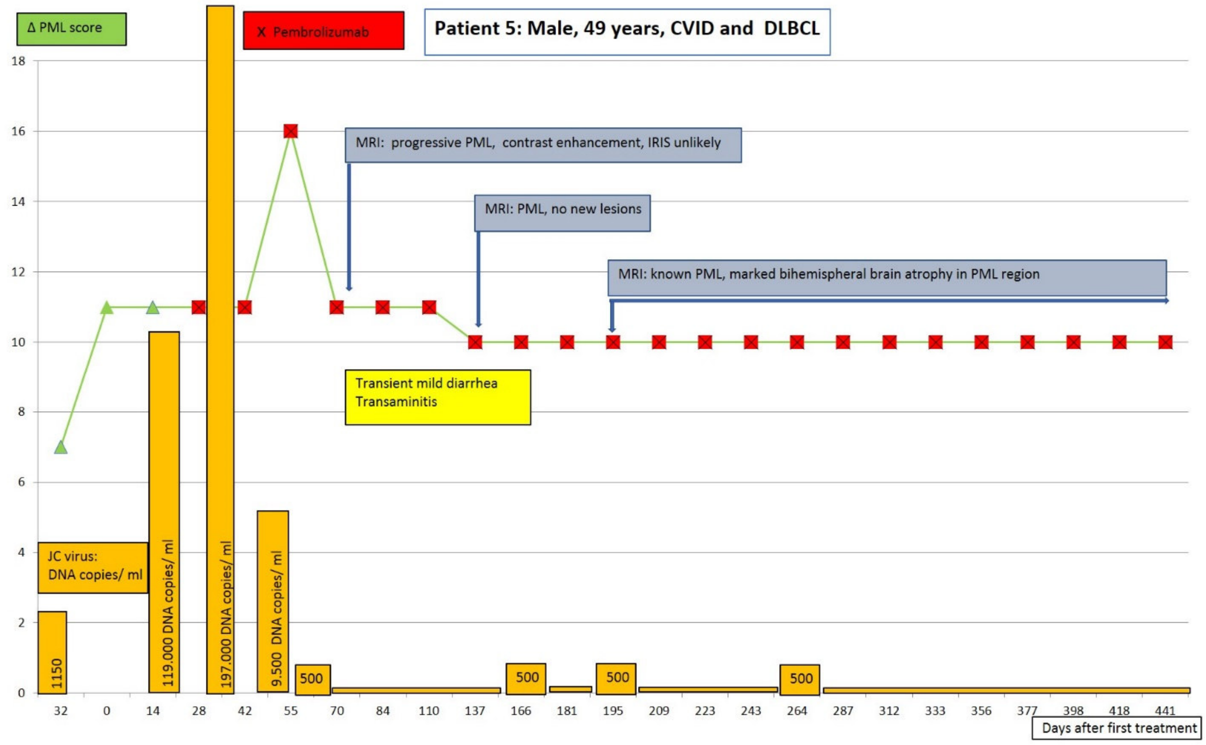

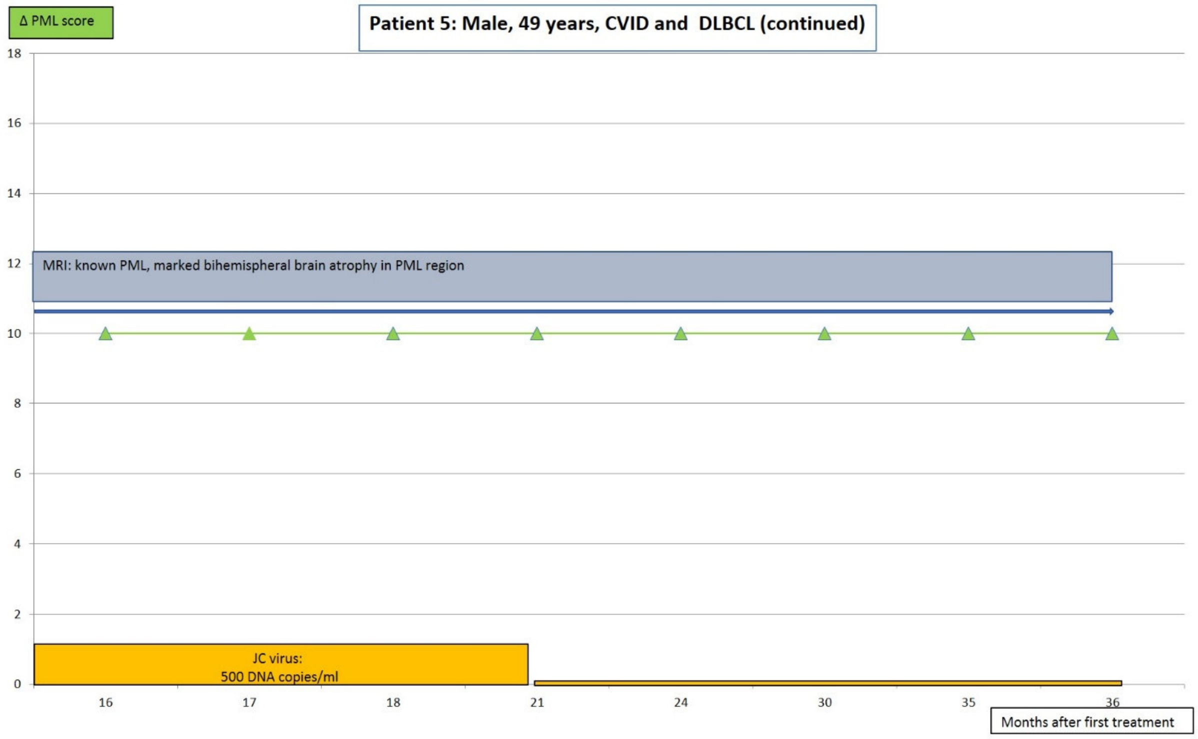

Figure S5: Clinical course of Patient 5

The PML Score is a clinical score comprised of the NIH stroke scale in addition to assessing vestibular function, dysphagia, executive function and memory previously proposed by Gasnault *et al.* [3] ranges are from 0 (no symptoms) to 60 (severe deficit)

Supplemental references:

1. Hulstaert, F., et al., *Age-related changes in human blood lymphocyte subpopulations. II. Varying kinetics of percentage and absolute count measurements.* Clin Immunol Immunopathol, 1994. **70**(2): p. 152-8.
2. Schatorje, E.J., et al., *Age-matched reference values for B-lymphocyte subpopulations and CVID classifications in children.* Scand J Immunol, 2011. **74**(5): p. 502-10.
3. Gasnault, J., et al., *Improved survival of HIV-1-infected patients with progressive multifocal leukoencephalopathy receiving early 5-drug combination antiretroviral therapy.* PLoS One, 2011. **6**(6): p. e20967.
